# Effectiveness of Regdanvimab Treatment in High-Risk COVID-19 Patients to Prevent Progression to Severe Disease

**DOI:** 10.3389/fimmu.2021.772320

**Published:** 2021-11-23

**Authors:** Ji Yeon Lee, Jee Young Lee, Jae-Hoon Ko, Miri Hyun, Hyun Ah Kim, Seongcheol Cho, Yong Dae Lee, Junghoon Song, Seunghwan Shin, Kyong Ran Peck

**Affiliations:** ^1^ Division of Infectious Diseases, Department of Internal Medicine, Keimyung University Dongsan Hospital, Keimyung University School of Medicine, Daegu, South Korea; ^2^ Department of Internal Medicine, Seoul Red Cross Hospital, Seoul, South Korea; ^3^ Division of Infectious Diseases, Department of Medicine, Samsung Medical Center, Sungkyunkwan University School of Medicine, Seoul, South Korea

**Keywords:** Regdanvimab, monoclonal antibody, COVID-19, progression, outcome

## Abstract

**Objective:**

To evaluate clinical effectiveness of regdanvimab, a monoclonal antibody agent for treating coronavirus 2019 (COVID-19).

**Methods:**

A retrospective cohort study was conducted at two general hospitals during the study period of December 2020 to May 2021. Mild COVID-19 patients with risk factors for disease progression admitted to the hospitals within seven days of symptom onset were enrolled and followed until discharge or referral. Multivariate analyses for disease progression were conducted in the total and propensity score (PS)-matched cohorts.

**Results:**

A total of 778 mild COVID-19 patients were included and classified as the regdanvimab (n = 234) and supportive care (n = 544) groups. Significantly fewer patients required O_2_ supplementation *via* nasal prong in the regdanvimab group (8.1%) than in the supportive care group (18.4%, *P* < 0.001). The decreased risk for O_2_ support by regdanvimab treatment was noticed in the multivariate analysis of the total cohort (HR 0.570, 95% CI 0.343–0.946, *P* = 0.030), but it was not statistically significant in the PS-matched cohort (*P* = 0.057). Progression to severe disease was also significantly lower in the regdanvimab group (2.1%) than in the supportive care group (9.6%, *P* < 0.001). The significantly reduced risk for progression to severe disease by regdanvimab treatment was observed in the analysis of both the total cohort (HR 0.262, 95% CI 0.103–0.667, *P* = 0.005) and PS-matched cohort (HR 0.176, 95% CI 0.060–0.516, *P* = 0.002). Potential risk factors for progression were investigated in the supportive care group and SpO_2_ < 97% and CRP elevation >1.5 mg/dL were common risk factors for O_2_ support and progression to severe disease. Among the patients with any of these factors, regdanvimab treatment was associated with decreased risk for progression to severe disease with slightly lower HR (HR 0.202, 95% CI 0.062–0.657, *P* = 0.008) than that of the total cohort.

**Conclusion:**

Regdanvimab treatment was associated with a decreased risk of progression to severe disease.

## Introduction

The coronavirus disease 2019 (COVID-19) pandemic is ongoing and has caused more than four million deaths as of October 2021 (1). Among the therapeutic agents tested against COVID-19, neutralizing monoclonal antibody (mAb) agents against severe acute respiratory syndrome coronavirus 2 (SARS-CoV-2) were found to decrease viral loads and prevent disease progression of mild COVID-19 (2–9). On September 2021, US National Institutes of Health recommended use of anti-SARS-CoV-2 mAb regimens, including casirivimab/imdevimab (Regeneron Pharmaceuticals Inc., NY, USA), bamlanivimab/etesevimab (Eli Lilly and Company, IN, USA), and sotrovimab (GlaxoSmithKline LLC, NC, USA), to treat non-hospitalized patients with mild to moderate COVID-19 who are at high risk of clinical progression (10). In the phase III trial, casirivimab/imdevimab decreased viral load faster than placebo, and COVID-19-related hospitalization or death from any causes were significantly reduced both in 2400mg and 1200mg arm (relative risk reduction of 71.3% and 70.4%, respectively) (7). Bamlanivimab/etesevimab also exhibited significant reduction of COVID-19-related hospitalization or death from any causes (relative risk difference, 70%) (9), and sotrovimab reduced COVID-19 progression risk by 85% (8).

Regdanvimab (CT-P59, Celltrion Inc, Incheon, Republic of Korea), a mAb agent against SARS-CoV-2, was approved by the Korea Ministry of Food and Drug Safety for the treatment of mild COVID-19 patients with risk factors for progression on February 5, 2021 based on the results of *in-vitro* study and the interim data of a phase II/III clinical trial (6, 11), and was reviewed by European Medicines Agency on March 2, 2021 for the support of national decisions on early use (12). In that trial, the incidence of severe COVID-19 cases requiring inpatient treatment was reduced by 54% among all COVID-19 patients and 68% among patients with moderate COVID-19 older than age 50. The time for clinical recovery was 5.4 days in the regdanvimab group, which was reduced by 3.4 days compared to 8.8 days in the placebo group (13). The approval in Korea was conditioned on the success of a phase III clinical trial, which was reported to meet its endpoints in June 2021 (14). To evaluate the clinical response to regdanvimab in the real world, we conducted a retrospective cohort study evaluating the pre- and post-periods of regdanvimab treatment.

## Methods

### Study Design and Population

This retrospective cohort study was conducted at two general hospitals designated for the care of mild and moderate COVID-19 patients between December 2020 and May 2021. The diagnosis of COVID-19 was made using the real-time polymerase chain reaction (RT-PCR) test for SARS-CoV-2. During the study period, most of mild COVID-19 patients were hospitalized at general COVID-19 designated hospitals, and worsening COVID-19 patients with O_2_ requirements of more than 5L per min *via* nasal prong or facial mask were referred to tertiary care centers. Regdanvimab was administered intravenously with the dose of 40mg/kg during hospitalization. Because regdanvimab was approved for administration within seven days of symptom onset, mild COVID-19 patients with any risk factors for disease progression who were admitted to the hospitals within seven days of symptom onset were screened. Mild COVID-19 was defined as COVID-19 patients who did not require O_2_ supplement at admission (SpO_2_ > 94% in room air). The risk factors for disease progression were 1) age ≥ 60 years, 2) cardiovascular disease, 3) chronic respiratory disease, 4) diabetes mellitus, 5) hypertension, and 6) radiologic evidence of pneumonia. Patients without any COVID-19 related symptoms, those without risk factors for progression, those admitted more than seven days after symptom onset, those referred to other hospitals before disease progression or recovery, and those who received regdanvimab more than seven days after symptom onset were excluded from the cohort. Attending physicians of both hospitals prescribed regdanvimab for the indicated patients after the drug became available on February 2021. Most of COVID-19 patients admitted from February to May 2021 received regdanvimab treatment if indicated, while those admitted from December 2020 to February 2021 did not receive the drug. There was an overlap period on February 2021. The enrolled patients were classified into the regdanvimab group or the supportive care group, and the clinical outcomes of the patients were followed until the day of discharge or referral. This study was approved by the Institutional Review Board of Samsung Medical Center (IRB no. 2021-07-079).

### Data Collection and Outcome Assessment

Baseline characteristics and epidemiologic information were collected from the electronic medical records. Clinical status at admission was evaluated using SpO_2_, radiologic evidence for pneumonia, complete blood count, chemistry profile, and C-reactive protein (CRP) levels. The initial cycle threshold (Ct) values of the RT-PCR at diagnosis (nasopharyngeal swab) were also collected. Ordinal disease severity scores were used to evaluate prognosis (**Supplementary Materials**) (15). The primary endpoints assessed were requiring O_2_ support *via* nasal prong (severity score 3) and a composite outcome indicating progression to severe disease (severity scores 4 to 8, including referral to a tertiary care hospital due to increasing O_2_ requirements). Requiring other treatment modalities including remdesivir, steroids, and antibiotics, and the length of hospital stay among patients who recovered without referral were compared as secondary outcomes.

### Statistical Analysis

To compare clinical factors, either the Student’s *t*-test or Mann–Whitney U test was used for continuous variables, and the Chi-square or Fisher’s exact test was used for categorical variables. The Kaplan-Meier method and long-rank test was used to calculate the 21-day probability of disease progression. Cox proportional hazard models were used to evaluate potential risk factors for disease progression within 21 days. All collected factors relevant to outcomes were evaluated in univariate analyses, and statistically significant factors were included in the multivariate analyses. When a continuous variable was statistically significant in the univariate analysis, it was converted into a categorical variable using interquartile ranges, the receiver operating characteristic (ROC) curve, or known normal limits, and the variable with the highest hazard ratio was included in the multivariate analysis. We used a propensity score (PS) matching method with the nearest neighbor matching algorithm and a 1:1 ratio without replacement to adjust potential confounders (16). A logistic regression analysis was performed to calculate the PS in a logistic model, and prognostic covariates reported from previous reports and those identified from the present cohort were included in that PS model (**Supplementary Materials**) (17–19). All *P*-values were two-tailed, and those <0.05 were considered statistically significant. IBM SPSS Statistics version 20.0 (IBM, Armonk, NY, USA) and R software (version 4.1.0 with packages; the R Project for Statistical Computing, Vienna, Austria) were used for all statistical analyses.

## Results

### Baseline Characteristics of Mild COVID-19 Patients With Risk Factors for Progression

**Figure 1:**
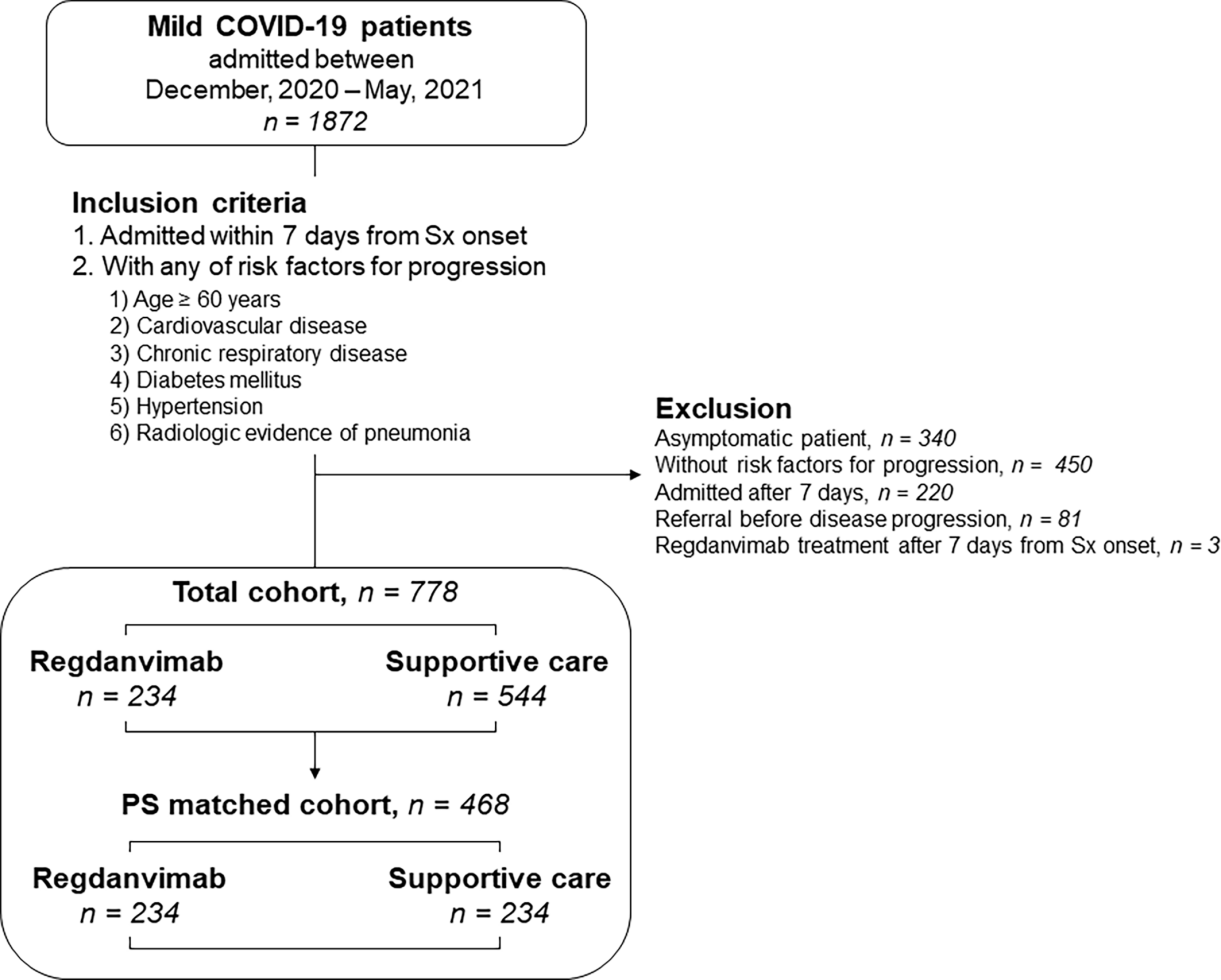
Patient selection for the retrospective cohort. Mild COVID-19 patients admitted to two hospitals designated for COVID-19 patient care were screened. High-risk patients who were admitted within 7 days of symptom onset were included. The effects of regdanvimab treatment were evaluated using multivariate analyses in the total cohort and in propensity score–matched cohorts developed to adjust for potential confounders. COVID-19, coronavirus disease 2019; Sx, symptom; PS, propensity score.

During the study period, 1872 mild COVID-19 patients were admitted to the two general hospitals designated for COVID-19 patient care (**Figure 1**). After excluding 1094 patients, 778 patients with risk factors for progression to severe disease who were admitted within seven days of symptom onset were included. The patients were classified into regdanvimab (n = 234) and supportive care (n = 544) groups. The supportive care group were diagnosed from November 26, 2020 to February 28, 2021, while the regdanvimab group were diagnosed from February 11 to May 31, 2021. The baseline characteristics of the patients are presented in **Table 1**. Patients in the regdanvimab group were younger (51.8 ± 14.3 years) than those in the supportive care group (56.2 ± 15.3 years, *P* < 0.001). Patients in the regdanvimab group were admitted earlier (2.8 ± 2.0 days from symptom onset) than those in the supportive care group (3.5 ± 2.2 days, *P* < 0.001). The average values of the initial laboratory tests were within normal ranges, except for CRP (1.6 ± 2.6 mg/dL). The CRP level was significantly lower in the regdanvimab group (1.2 ± 1.7 mg/dL) than in the supportive care group (1.7 ± 2.9 mg/dL, *P* = 0.001).

**Table 1:** Baseline characteristics of the regdanvimab and supportive care groups.

Variables	Regdanvimab	Supportive care	*P* value
	(n = 234)	(n = 544)	
**Demographics**			
Age, years	51.8 ± 14.3	56.2 ± 15.3	<0.001
Male sex	130 (55.6)	267 (49.1)	0.101
BMI	25.2 ± 3.6	24.8 ± 3.6	0.112
Sx onset to admission, days	2.8 ± 2.0	3.5 ± 2.2	<0.001
**Initial presentation**			
Initial Ct value (NP swab, RdRp)	19.2 ± 6.4	19.9 ± 5.7	0.160
Pneumonia	168 (71.8)	354 (65.1)	0.068
SIRS	47 (20.1)	95 (17.5)	0.418
SpO_2_	97.2 ± 1.0	97.3 ± 1.2	0.748
**Initial laboratory tests**			
WBC count,/μL	4749.4 ± 1505.4	4812.3 ± 1535.9	0.599
Lymphocyte count,/μL	1202.1 ± 460.9	1264.3 ± 720.8	0.224
Platelet count, x10^3^/μL	199.7 ± 61.3	191.8 ± 66.0	0.116
Total bilirubin, mg/dL	0.62 ± 0.3	0.59 ± 0.2	0.155
Albumin, g/dL	4.5 ± 0.3	4.4 ± 0.3	0.002
AST, IU/L	32.7 ± 18.5	33.3 ± 19.6	0.704
ALT, IU/L	33.7 ± 23.6	32.9 ± 24.9	0.668
BUN, mg/dL	13.5 ± 4.0	14.4 ± 4.9	0.004
Creatinine, mg/dL	0.8 ± 0.2	0.9 ± 0.3	0.287
CPK, IU/L	128.3 ± 185.7	127.5 ± 165.2	0.953
LDH, IU/L	388.7 ± 100.9	398.0 ± 121.3	0.265
CRP, mg/dL	1.2 ± 1.7	1.7 ± 2.9	0.001
Underlying diseases^*^			
Cardiovascular disease	22 (9.4)	54 (9.9)	0.896
Respiratory disease	8 (3.4)	35 (6.4)	0.122
Diabetes mellitus	40 (17.1)	91 (16.7)	0.917
Hypertension	79 (33.8)	182 (33.5)	0.934
Liver disease	4 (1.7)	7 (1.3)	0.647
Renal disease	1 (0.4)	3 (0.6)	0.824
Charlson Comorbidity Index	0 (0–1)	0 (0–1)	0.176

Data are expressed as the number (%) of patients, mean ± SD, or median (IQR) unless indicated otherwise. ^*^There were no immunocompromised patients, such as hematology/oncology patients, organ transplant recipients, or HIV-infected patients.

BMI, body mass index; Sx, symptom; Ct, cycle threshold; NP, nasopharyngeal; RdRp, RNA-dependent RNA polymerase; SIRS, systemic inflammatory response syndrome; SpO_2_, saturation of percutaneous oxygen; WBC, white blood cell; AST, aspartate aminotransferase; ALT, alanine aminotransferase; BUN, blood urea nitrogen; CPK, creatine phosphokinase; LDH, lactate dehydrogenase; CRP, C-reactive protein; HIV, human immunodeficiency virus.

### Treatment and Outcomes of the Cohort Patients

The treatment and outcomes of the regdanvimab and supportive care groups are summarized in **Table 2**. Patients in the regdanvimab group received regdanvimab treatment an average of 4.0 days after symptom onset and 2.2 days after admission. No patient in the regdanvimab group received remdesivir, but three in the supportive care group did (0.6%). Significantly less patients received steroid treatment in the regdanvimab group (9.8%) than in the supportive care group (19.1%, *P* = 0.001). Other immune modulators, such as baricitinib or tocilizumab, were not administered for the study population and no one participated in other clinical trials for therapeutics. No patient in the regdanvimab group received antibiotics treatment, but 73 patients (13.4%) in the supportive care group did (*P* < 0.001).

**Table 2:** Treatment and outcomes of the regdanvimab and supportive care groups.

Variables	Regdanvimab	Supportive care	*P* value
	(n = 234)	(n = 544)	
**Regdanvimab**			
Regdanvimab treatment	234 (100.0))	0 (0.0)	NA
Interval from symptom onset to regdanvimab, days	4.0 ± 1.8	NA	NA
Interval from admission to regdanvimab, days	2.2 ± 1.5	NA	NA
**Remdesivir, steroids, and antibiotics** ^*^			
Remdesivir treatment	0 (0.0)	3 (0.6)	0.255
Interval from admission to remdesivir, days	NA	5.7 ± 4.0	NA
Steroid treatment	23 (9.8)	104 (19.1)	0.001
Interval from admission to steroids, days	2.8 ± 2.4	4.0 ± 3.5	0.119
Antibiotic treatment	0 (0.0)	73 (13.4)	<0.001
Interval from admission to antibiotics, days	NA	4.3 ± 3.7	NA
**Outcome measures**			
**O_2_ supplementation *via* nasal prong**	19 (8.1)	100 (18.4)	<0.001
Interval from admission to nasal prong, days	2.0 (2.0–4.0)	3.0 (2.0–5.0)	0.129
**Composite outcome for progression to severe disease**	5 (2.1)	52 (9.6)	<0.001
Interval from admission to composite outcome, days	5.0 (2.5–7.5)	5.0 (3.0–8.0)	0.691
O_2_ supplement *via* facial mask	1 (0.4)	12 (2.2)	0.076
Interval from admission to facial mask, days	10.0 (10.0–10.0)	6.5 (5.0–7.8)	0.154
O_2_ supplement *via* HFNC	0 (0.0)	8 (1.5)	0.062
Interval from admission to HFNC, days	NA	7.0 (6.0–8.8)	NA
Referral to tertiary care center**^†^**	5 (2.1)	49 (9.0)	<0.001
Interval from admission to referral, days	5.0 (2.5–7.5)	5.0 (3.0–8.0)	0.664
**Live discharge after recovery without referral**	229 (97.9)	494 (90.8)	<0.001
Interval from admission to discharge, days	11.0 (9.0–12.5)	12.0 (10.0–15.0)	<0.001
**In-hospital mortality during follow-up period** ^‡^	0 (0.0)	1 (0.2)	0.512

Data are expressed as the number (%) of patients or mean ± SD unless indicated otherwise. ^*^Other immune modulators, such as baricitinib or tocilizumab, were not administered for the study population and no one participated in other clinical trials for therapeutics. **^†^** ^‡^One patient in the supportive care group could not be referred to a tertiary care center due to insufficient capacity; that patient received mechanical ventilation support and died. ^‡^Outcome of patient was followed until discharge or referral. Final outcomes of referred patients were not collected.

NA, not applicable; HFNC, high flow nasal cannula.

**Figure 2:**
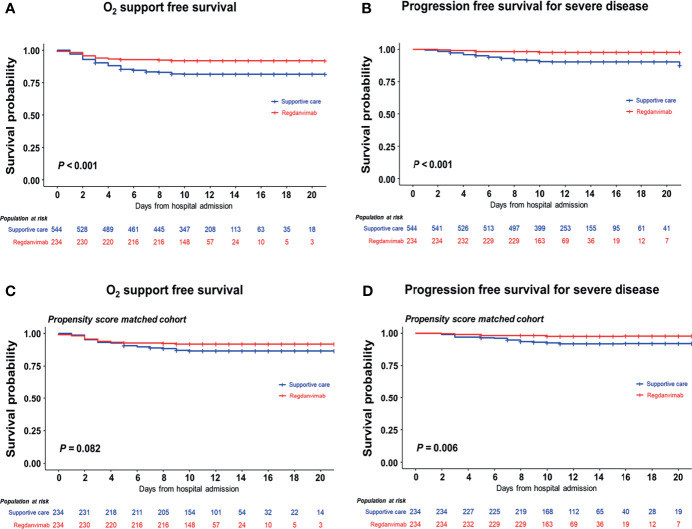
Progression-free survival analysis in the total and propensity score–matched cohorts. The 21-day probabilities for composite outcomes 1 **(A)** and 2 **(B)** were evaluated in the total cohort, and the regdanvimab group showed clinical benefit for both outcomes. Statistically significant benefits were also found in the propensity score–matched cohort for composite outcomes 1 **(C)** and 2 **(D)**.

After admission, significantly less patients required O_2_ supplementation *via* nasal prong in the regdanvimab group (8.1%) than in the supportive care group (18.4%, *P* < 0.001). When O_2_ support free survival was calculated using the Kaplan-Meier method, significantly fewer patients required O_2_ support in the regdanvimab group than in the supportive care group (*P* < 0.001, **Figure 2A**). Significantly less patients progressed to severe disease in the regdanvimab group (2.1%) than in the supportive care group (9.6%, *P* < 0.001). When progression-free survival for severe disease was calculated using the Kaplan-Meier method, significantly fewer patients progressed to severe disease in the regdanvimab group than in the supportive care group (*P* < 0.001, **Figure 2B**). Significantly more patients were discharged after recovery without referral to tertiary care centers in the regdanvimab group (97.9%) than in the supportive care group (90.8%, *P* < 0.001), and the hospital stays were also shorter in the regdanvimab group (median 11.0 days, interquartile range (IQR) 9.0–12.5 days) than the supportive care group (median 12.0 days, IQR 10.0–15.0 days, *P* < 0.001).

### Univariate and Multivariate Analyses for 21-Day Disease Progression Probability in Total Cohort

To identify potential confounding factors for O_2_ support *via* nasal prong and progression to severe disease, univariate and multivariate analyses were conducted (**Supplementary Tables 1**, **2**). In the multivariate analysis, regdanvimab treatment was significantly associated with a decreased risk for O_2_ support *via* nasal prong (HR 0.570, 95% CI 0.343–0.946, *P* = 0.030; **Table 3**). Age ≥ 70 years, SpO_2_ < 97%, thrombocytopenia, creatinine level, CRP elevation > 1.5 mg/dL, cardiovascular disease, and hypertension were associated with an increased risk for O_2_ support. The risk for progression to severe disease was also significantly decreased with regdanvimab treatment (HR 0.262, 95% CI 0.103–0.667, *P* = 0.005). SpO_2_ < 97% and CRP elevation > 1.5 mg/dL were associated with an increased risk for progression to severe disease.

**Table 3:** Multivariate analysis of 21-day disease progression probability.

Factors for disease progression	O_2_ support		Progression to severe disease	
	HR (95% CI)	*P* value	HR (95% CI)	*P* value
Age ≥ 70 years	2.024 (1.322–3.099)	0.001	1.106 (0.576–2.123)	0.763
Male sex	0.947 (0.609–1.472)	0.808	1.560 (0.792–3.074)	0.199
BMI	1.037 (0.985–1.091)	0.166		
BMI ≥ 25			1.555 (0.889–2.721)	0.122
Sx onset to admission, days	1.062 (0.963–1.170)	0.230		
SpO_2_ < 97%^*^	2.970 (2.018–4.372)	<0.001	2.697 (1.545–4.708)	<0.001
Neutrophil > 3500/μL	1.321 (0.878–1.990)	0.182	1.283 (0.717–2.297)	0.402
Thrombocytopenia (<150 x10^3^/μL)	2.103 (1.402–3.155)	<0.001	1.720 (0.958–3.087)	0.069
Albumin < 4.0 g/dL	1.379 (0.851–2.234)	0.192	1.574 (0.750–3.303)	0.230
Creatinine, mg/dL	2.394 (1.141–5.023)	0.021	2.028 (0.681–6.041)	0.204
CPK, IU/L			1.001 (1.000–1.002)	0.199
CPK elevation (>250 IU/L)	1.144 (0.659–1.988)	0.632		
LDH elevation (>400 IU/L)	1.326 (0.880–1.999)	0.177	0.920 (0.513–1.650)	0.780
CRP elevation (>1.5 mg/dL)	2.742 (1.779–4.225)	<0.001	2.414 (1.292–4.509)	0.006
Cardiovascular disease	1.703 (1.020–2.845)	0.042		
Hypertension	1.564 (1.054–2.322)	0.027	1.403 (0.792–2.486)	0.246
***Regdanvimab treatment***	0.570 (0.343–0.946)	0.030	0.262 (0.103–0.667)	0.005

^*^Median value of total cohort.

HR, hazard ratio; CI, confidence interval; BMI, body mass index; Sx, symptom; SpO_2_, saturation of percutaneous oxygen; CPK, creatine phosphokinase; LDH, lactate dehydrogenase; CRP, C-reactive protein.

To investigate potential risk factors for progression of the study population in the absence of the regdanvimab effect, analyses were conducted in the supportive care group (**Supplementary Tables 3**, **4**). Among the variables found to be significant in the univariate analyses, SpO_2_ < 97% and CRP elevation >1.5 mg/dL remained significant risk factors in the multivariate analyses for O_2_ support and progression to severe disease. When the total cohort was classified into a higher-risk group (patients with SpO_2_ < 97% or CRP > 1.5 mg/dL, n = 319) and a lower-risk group (patients without either of those two risk factors, n = 459), a significantly higher proportion of the patients in the higher-risk group required O_2_ supplementation (28.5%) and progressed to severe disease (14.4%), compared with those in the low-risk group (6.1%, and 2.4%, respectively, both *P* < 0.001). In the multivariate analyses of the high-risk group, regdanvimab treatment was associated with decreased risk for progression to severe disease with slightly lower HR (HR 0.202, 95% CI 0.062–0.657, *P* = 0.008) than that of the total cohort (**Supplementary Table 5**).

To identify risk factors for regdanvimab failure, analyses were conducted in the regdanvimab group (**Supplementary Tables 6**, **7**). In the multivariate analysis, age ≥ 60 years, time from symptom onset to admission, SpO_2_ < 97%, and thrombocytopenia were associated with an increased risk for O_2_ support, but only systemic inflammatory response syndrome (SIRS) was associated with an increased risk for progression to severe disease.

### Analysis of a PS-Matched Cohort

A PS-matched cohort was developed with 1:1 ratio, and 234 patients in the supportive care group were matched to 234 patients in the regdanvimab group. The baseline characteristics of the matched cohort were statistically balanced (**Supplementary Table 8**). In the multivariate analysis, regdanvimab treatment was not significantly associated with a decreased risk for O_2_ support *via* nasal prong (HR 0.548, 95% CI 0.301–0.999, *P* = 0.050), but it was significantly associated with a decreased risk for progression to severe disease (HR 0.176, 95% CI 0.060–0.516, *P* = 0.002; **Table 4** and **Supplementary Tables 9**, **10**). These findings were also observed in the survival analysis using the Kaplan-Meier method (**Figures 2C, D**).

**Table 4:** Multivariate analysis of 21-day disease progression probability of the PS-matched cohort.

Factors for disease progression	O_2_ support		Progression to severe disease	
	HR (95% CI)	*P* value	HR (95% CI)	*P* value
Age ≥ 70 years	1.120 (0.507-2.476)	0.779		
Sx onset to admission, days	1.233 (1.052–1.445)	0.010		
SIRS	1.699 (0.885–3.262)	0.111		
SpO_2_ < 97%^*^	3.847 (2.111–7.011)	<0.001	2.829 (1.186–6.747)	0.019
Thrombocytopenia (<150 x10^3^/μL)	2.864 (1.563–5.250)	<0.001	1.909 (0.814–4.478)	0.003
Albumin < 4.0 g/dL	1.875 (0.752–4.679)	0.178		
BUN elevation (>19 mg/dL)	1.490 (0.628–3.535)	0.366		
LDH, IU/L			1.004 (1.001–1.008)	0.025
LDH elevation (>400 IU/L)	1.813 (0.984–3.339)	0.056		
CRP elevation (>5 mg/dL)	3.613 (1.569–8.319)	0.003	5.881 (1.843–18.771)	0.003
Cardiovascular disease	3.456 (1.617–7.385)	0.001		
Diabetes mellitus	1.684 (0.874–3.243)	0.119		
Hypertension	1.546 (0.853–2.802)	0.151		
***Regdanvimab treatment***	0.548 (0.301–0.999)	0.050	0.176 (0.060–0.516)	0.002

^*^Median value of total cohort.

HR, hazard ratio; CI, confidence interval; Sx, symptom; SpO_2_, saturation of percutaneous oxygen; BUN, blood urea nitrogen; LDH, lactate dehydrogenase; CRP, C-reactive protein.

## Discussion

Passive immunization using mAb products require healthcare resources for intravenous administration to mild COVID-19 patients (10). To overcome such limitation, casirivimab/imdevimab added indication for subcutaneous injection based on a phase I trial (20). In the Republic of Korea, mAb agents are practically applicable to mild COVID-19 patients with risk factors for progression because those patients are managed at general hospitals where intravenous administration is available (21, 22). After the conditioned approval of regdanvimab on February 17, 2021, several COVID-19-designated hospitals began actively administering regdanvimab to indicated patients. To date, regdanvimab has been widely administered in Korean COVID-19 designated hospitals, but the data from clinical trials of regdanvimab have not been published as a full scientific article yet (13). As evaluation of the clinical efficacy of treatment modalities in a real-world setting would be especially necessary for drugs under emergency use authorizations (4), we conducted the present retrospective cohort study for scientific background for COVID-19 management.

Of note, in the multivariate analyses of the total cohort and PS-matched cohort, regdanvimab treatment was significantly associated with a decreased risk of progression to severe disease. Although a reduced risk for requiring O_2_ supplementation was not statistically significant in the PS-matched cohort, a tendency favoring the regdanvimab group was noticed, consistent with the analysis of the total cohort. The need for other treatment modalities, including remdesivir, steroids, and antibiotics, was significantly lower in the regdanvimab group, and the length of hospital stay among patients who recovered without referral was also shorter in the regdanvimab group than in the supportive care group. These findings consistently indicate that regdanvimab treatment offers clinical benefit for high-risk patients. Because most mild to moderate COVID-19 patients of the Republic of Korea are managed at designated general hospitals, these primary outcomes of the present study are not identical with those of clinical trials conducted for non-hospitalized COVID-19 patients (4, 7, 9). The clinical status of COVID-19 patients who require O_2_ supplementation in the present study would be similar or slightly more severe than those who require hospitalization in those trials, though indications for hospitalization were not clearly presented (4, 7, 9). Since those clinical trials did not evaluated progression to severe disease as an outcome value, the finding of the present study would additionally support effectiveness of mAb agents for mild to moderate COVID-19 patients.

However, the progression rate in this study population was low (18.4% for O_2_ supplement and 9.6% for progression to severe disease in the supportive care group), because regdanvimab treatment is approved for early COVID-19 patients who have relatively broad risk factors for disease progression. The proportion of COVID-19-related hospitalization was much lower in the clinical trials of other mAb agents for non-hospitalized patients (control arms, 3.2–7.0%) (7, 9). To figure out whether a high-risk subgroup might gain more benefit from regdanvimab treatment, we investigated the common risk factors for progression in our analyses of the total cohort and supportive care group. Among the various clinical variables, SpO_2_ < 97% and CRP level > 1.5 mg/dL were common risk factors for progression, and the progression rate in the higher-risk sub-group (of patients with either of those factors) was 28.5% for O_2_ supplementation and 14.4% for progression to severe disease. Meanwhile, in the lower-risk sub-group (of patients without any of these two factors), progression rates to O_2_ supplement and to severe disease were 6.1% and 2.4% respectively, which were similar to those treated with regdanvimab. Adding those factors to the treatment guidelines could increase the cost-effectiveness, but a certain proportion of patients would lose their treatment opportunity. The appropriate indication for mAb treatment thus needs to be adjusted based on the outbreak situation and healthcare resources.

Although only five patients in the regdanvimab group (2.1%) progressed to severe disease, we also conducted multivariate analyses to identify the risk factors for regdanvimab failure. It was difficult to find consistent factors between the two primary endpoints, but the statistically significant factors of late admission, decreased oxygenation, thrombocytopenia, and SIRS might be associated with progressed disease. The time interval between symptom onset to admission or regdanvimab treatment was associated with O_2_ supplementation, consistent with previous cohort study conducted at USA (4), but administration time interval was not associated with progression to severe disease in the present cohort. These findings suggest that early administration of regdanvimab would be important, but other unmeasured factors may have stronger association with regdanvimab failure. Since potential virulence factors associated with viral mutation was not evaluated in the present analysis, a larger cohort study with viral sequencing needs to be conducted to clearly elucidate the factors involved in regdanvimab failure.

The present study has several limitations. First, we retrospectively evaluated patients before and after regdanvimab became available. Even though the management in the outbreak setting could be different, it is less likely because study population were admitted at early time point after symptoms onset and managed at the same hospital. During the study period, management for mild COVID-19 patients did not change, and the community-based spread of SARS-CoV-2 variants of concerns (VOCs) was not significant in the Republic of Korea (23). The two hospitals participated in the present study have been dedicated for mild and moderate COVID-19 patient care since the first (March 2020) and second (June 2020) domestic outbreak, respectively, and the medical resources and management protocols were well-stabilized before the start of present study (December 2020). Nevertheless, basic demographic factors such as age, sex, and underlying disease could be variable according to the outbreak situation and seasons. To overcome that limitation, we enrolled a control group more than two times larger than the regdanvimab group in a short period and performed multivariate analyses and PS matching. Second, the cohort study was conducted in two general hospitals, and outcomes after patients were referred to tertiary care centers could not be investigated. During hospitalization at these general hospitals, only one patient in the supportive care group died who could not be referred to a tertiary care center due to insufficient capacity. A large, nation-wide cohort study is needed to evaluate the final outcomes of patients whose disease progressed despite regdanvimab treatment. Last, although the study patients were confirmed with COVID-19 with SARS-CoV-2 RT-PCR, whole genome sequencing (WGS) to detect viral mutation was not performed. Although the effectiveness of mAbs could be different against VOCs, VOCs were not dominant during the study period in the Republic of Korea. The healthcare authority selectively performed WGS to detect viral mutation for risk groups such as immigrants from VOC-endemic countries and individuals exposed to VOCs. In the present cohort, only one case in the regdanvimab group was reported to be infected with alpha variant. Although he progressed to severe disease despite regdanvimab treatment, it was difficult to interpret the impact of a single case of VOC. Follow-up studies against VOCs need to be conducted during a VOC-dominant outbreak period with sequencing of entire study population.

In conclusion, in a retrospective cohort study evaluating high-risk COVID-19 patients, regdanvimab treatment within seven days of symptom onset was associated with decreased risk of progression to severe disease.

## Data Availability Statement

The original contributions presented in the study are included in the article/**Supplementary Material**. Further inquiries can be directed to the corresponding authors.

## Ethics Statement

The studies involving human participants were reviewed and approved by Samsung Medical Center. Written informed consent for participation was not required for this study in accordance with the national legislation and the institutional requirements.

## Author Contributions

JiL, JeL, J-HK, SS, and KP contributed to the conceptualization. JiL, JeL, J-HK, MH, HK, SC, YDL, JS, and SS, and KP contributed to the investigation. J-HK and SS contributed to the statistical analysis. KP contributed to the supervision. J-HK, SS, and KP contributed to the writing, review, and editing. All authors contributed to the article and approved the submitted version.

## Funding

This work was supported by Research Program funded by the Korea Disease Control and Prevention Agency (#2021-ER1906-00).

## Conflict of Interest

The authors declare that the research was conducted in the absence of any commercial or financial relationships that could be construed as a potential conflict of interest.

## Publisher’s Note

All claims expressed in this article are solely those of the authors and do not necessarily represent those of their affiliated organizations, or those of the publisher, the editors and the reviewers. Any product that may be evaluated in this article, or claim that may be made by its manufacturer, is not guaranteed or endorsed by the publisher.
