# Supplementary material for: Effectiveness of Regdanvimab Treatment in High-Risk COVID-19 Patients to Prevent Progression to Severe Disease
Source: Front Immunol. 2021 Nov 23;12:772320. doi: 10.3389/fimmu.2021.772320 (PMC8657590; doi:10.3389/fimmu.2021.772320)
Supplement: Supplementary file 1 [file DataSheet_1.docx]

**Supplementary materials**

***List of contents***

**Ordinal disease severity scores**

**Propensity score matching**

**Supplementary Table 1.** Univariate and multivariate analyses of the 21-day probability of progression to O2 support via nasal prong (total cohort, n = 778)

**Supplementary Table 2.** Univariate and multivariate analyses of the 21-day probability of progression to severe disease (total cohort, n = 778)

**Supplementary Table 3.** Univariate and multivariate analyses of the 21-day probability of progression to O2 support via nasal prong (supportive care group, n = 544)

**Supplementary Table 4.** Univariate and multivariate analyses of the 21-day probability of progression to severe disease (supportive care group, n = 544)

**Supplementary Table 5.** Univariate and multivariate analyses for 21-day probability of progression to severe disease (high risk group with additional risk factors, n = 319)

**Supplementary Table 6.** Univariate and multivariate analyses of the 21-day probability of progression to O2 support via nasal prong (regdanvimab group, n = 234)

**Supplementary Table 7.** Univariate and multivariate analyses of the 21-day probability of progression to severe disease (regdanvimab group, n = 234)

**Supplementary Table 8.** Baseline characteristics of the regdanvimab and supportive care groups in the PS-matched cohorts (n = 468)

**Supplementary Table 9.** Univariate and multivariate analyses of the 21-day probability of progression to O2 support via nasal prong (PS-matched cohorts, n = 468)

**Supplementary Table 10.** Univariate and multivariate analyses of the 21-day probability of progression to severe disease (PS-matched cohorts, n = 468)

**Ordinal disease severity scores**

- score 1 (no limitation of daily activities)

- score 2 (limitation of daily activities but no need for supplemental O_2_)

- score 3 (need for supplemental O_2_ via nasal prong)

- score 4 (need for supplemental O_2_ via facial mask)

- score 5 (need for high-flow supplemental O_2_ or noninvasive mechanical ventilation)

- score 6 (need for invasive mechanical ventilation)

- score 7 (multi-organ failure or the need for extracorporeal membrane oxygenation therapy)

- score 8 (death)

**Propensity score matching**

We used a propensity score (PS) matching method with the nearest neighbor matching algorithm and a 1:1 ratio without replacement to adjust potential confounders. A logistic regression analysis was performed to calculate the PS in a logistic model, and prognostic covariates reported from previous reports and those identified from the present cohort such as age, sex, symptom onset to admission, pneumonia, body mass index (BMI), SpO2, diabetes mellitus, lymphocyte count, platelet count, and albumin, blood urea nitrogen, lactate dehydrogenase, and CRP levels, were included in that PS model. We matched regdanvimab-treated and non-treated patients and used the absolute standardized mean difference (ASD) to determine the balance between the two groups before and after PS matching. ASDs between the two groups were set below 0.2 to determine whether the groups were well-balanced through matching. After confirming the adequacy of the PS matching, we performed Cox proportional hazards regression analyses for disease progression within 21 days in the PS-matched cohorts.


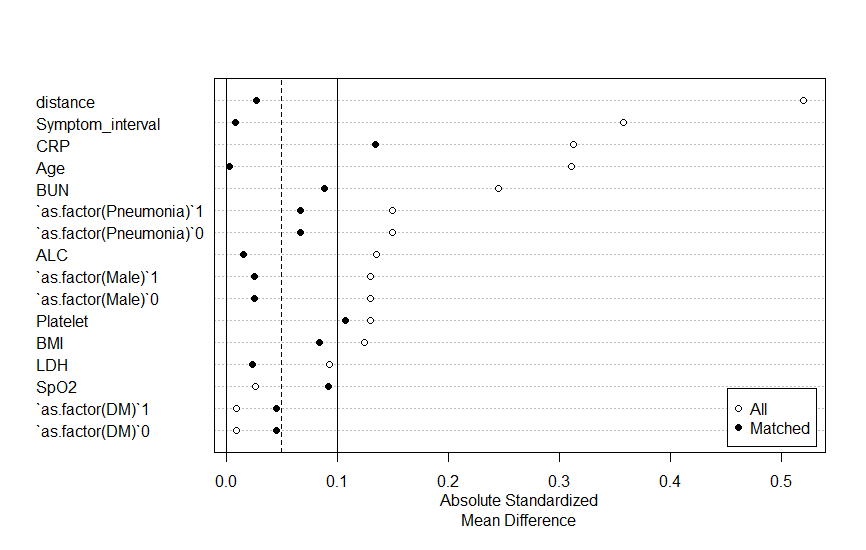


**ASD of total and matched cohort**

**Supplementary Table 1. Univariate and multivariate analyses of the 21-day probability of progression to O_2_ support via nasal prong (total cohort, n = 778)**

| **Variables** | ***Univariate analysis*** | |  | ***Multivariate analysis*** | |
| --- | --- | --- | --- | --- | --- |
|  | **HR (95% CI)** | ***P* value** |  | **HR (95% CI)** | ***P* value** |
| **Demographics** |  |  |  |  |  |
| Age, years | 1.045 (1.030–1.060) | <0.001 |  |  |  |
| *Age ≥ 60 years* | 2.717 (1.853–3.984) | <0.001 |  |  |  |
| *Age ≥ 70 years* | 3.297 (2.275–4.776) | <0.001 |  | 2.024 (1.322–3.099) | 0.001 |
| Male sex | 1.458 (1.011–2.103) | 0.044 |  | 0.947 (0.609–1.472) | 0.808 |
| BMI | 1.051 (1.002–1.101) | 0.040 |  | 1.037 (0.985–1.091) | 0.166 |
| *BMI ≥ 25* | 1.349 (0.942–1.932) | 0.103 |  |  |  |
| *BMI ≥ 30* | 0.971 (0.492–1.915) | 0.932 |  |  |  |
| Sx onset to admission, days | 1.183 (1.083–1.293) | <0.001 |  | 1.062 (0.963–1.170) | 0.230 |
| **Initial presentation** |  |  |  |  |  |
| Initial Ct value (NP swab, RdRp) | 1.006 (0.974–1.038) | 0.735 |  |  |  |
| Pneumonia | 0.947 (0.649–1.383) | 0.780 |  |  |  |
| SIRS | 1.511 (0.994–2.296) | 0.053 |  |  |  |
| SpO_2_ | 0.566 (0.487–0.658) | <0.001 |  |  |  |
| *SpO_2_ <97%* | 3.435 (2.397–4.922) | <0.001 |  | 2.970 (2.018–4.372) | <0.001 |
| **Initial laboratory tests** |  |  |  |  |  |
| WBC count, x10^3^/μL | 1.117 (1.007–1.239) | 0.036 |  |  |  |
| *WBC count > 5500 /μL* | 1.561 (1.064–2.289) | 0.023 |  |  |  |
| *Leukopenia (<4000 /μL)* | 0.751 (0.498–1.130) | 0.170 |  |  |  |
| Neutrophil count, x10^3^/μL | 1.198 (1.079–1.333) | 0.001 |  |  |  |
| *Neutrophil > 3500 /μL* | 1.826 (1.259–2.649) | 0.002 |  | 1.321 (0.878–1.990) | 0.182 |
| Lymphocyte count, x10^3^/μL | 0.632 (0.417–0.959) | 0.031 |  |  |  |
| *Lymphopenia (<1500/μL)* | 1.312 (0.908–1.895) | 0.148 |  |  |  |
| Platelet count, x10^3^/μL | 0.986 (0.982–0.989) | <0.001 |  |  |  |
| *Thrombocytopenia (<150 x10^3^/μL)* | 3.328 (2.321–4.771) | <0.001 |  | 2.103 (1.402–3.155) | <0.001 |
| Total bilirubin, mg/dL | 0.692 (0.319–1.502) | 0.352 |  |  |  |
| Albumin, g/dL | 0.209 (0.123–0.357) | <0.001 |  |  |  |
| *Albumin < 4.0 g/dL* | 3.246 (2.100–5.017) | <0.001 |  | 1.379 (0.851–2.234) | 0.192 |
| AST, IU/L | 1.005 (0.997–1.012) | 0.230 |  |  |  |
| ALT, IU/L | 0.999 (0.992–1.007) | 0.889 |  |  |  |
| BUN, mg/dL | 1.084 (1.053–1.115) | <0.001 |  |  |  |
| *BUN elevation (>19 mg/dL)* | 2.805 (1.854–4.243) | <0.001 |  |  |  |
| Creatinine, mg/dL | 5.140 (3.155–8.373) | <0.001 |  | 2.394 (1.141–5.023) | 0.021 |
| *Creatinine elevation (>1.2 mg/dL)* | 3.603 (2.181–5.952) | <0.001 |  |  |  |
| CPK, IU/L | 1.001 (1.000–1.001) | 0.020 |  |  |  |
| *CPK elevation (>250 IU/L)* | 2.239 (1.340–3.742) | 0.002 |  | 1.144 (0.659–1.988) | 0.632 |
| LDH, IU/L | 1.004 (1.002–1.005) | <0.001 |  |  |  |
| *LDH elevation (>400 IU/L)* | 2.353 (1.633–3.389) | <0.001 |  | 1.326 (0.880–1.999) | 0.177 |
| CRP, mg/dL | 1.199 (1.155–1.244) | <0.001 |  |  |  |
| *CRP elevation (>1.5 mg/dL)* | 5.217 (3.605–7.550) | <0.001 |  | 2.742 (1.779–4.225) | <0.001 |
| *CRP elevation (>5 mg/dL)* | 4.296 (2.810–6.569) | <0.001 |  |  |  |
| **Underlying diseases** |  |  |  |  |  |
| Cardiovascular disease | 2.021 (1.250–3.269) | 0.004 |  | 1.703 (1.020–2.845) | 0.042 |
| Respiratory disease | 1.496 (0.758–2.952) | 0.245 |  |  |  |
| Diabetes mellitus | 1.417 (0.917–2.189) | 0.116 |  |  |  |
| Hypertension | 2.135 (1.490–3.058) | <0.001 |  | 1.564 (1.054–2.322) | 0.027 |
| Liver disease | 1.189 (0.294–4.812) | 0.808 |  |  |  |
| Renal disease | 2.079 (0.290–14.893) | 0.466 |  |  |  |
| Charlson Comorbidity Index | 1.485 (1.177–1.874) | 0.001 |  |  |  |
| **Regdanvimab treatment** | 0.424 (0.260–0.693) | 0.001 |  | 0.570 (0.343–0.946) | 0.030 |

Abbreviations: HR, hazard ratio; CI, confidence interval; BMI, body mass index; Sx, symptom; Ct, cycle threshold; NP, nasopharyngeal; RdRp, RNA-dependent RNA polymerase; SIRS, systemic inflammatory response syndrome; SpO_2_, saturation of percutaneous oxygen; WBC, white blood cell; AST, aspartate aminotransferase; ALT, alanine aminotransferase; BUN, blood urea nitrogen; CPK, creatine phosphokinase; LDH, lactate dehydrogenase; CRP, C-reactive protein

**Supplementary Table 2. Univariate and multivariate analyses of the 21-day probability of progression to severe disease (total cohort, n = 778)**

| **Variables** | ***Univariate analysis*** | |  | ***Multivariate analysis*** | |
| --- | --- | --- | --- | --- | --- |
|  | **HR (95% CI)** | ***P* value** |  | **HR (95% CI)** | ***P* value** |
| **Demographics** |  |  |  |  |  |
| Age, years | 1.023 (1.004–1.042) | 0.020 |  |  |  |
| *Age ≥ 60 years* | 1.527 (0.903–2.584) | 0.114 |  |  |  |
| *Age ≥ 70 years* | 2.144 (1.221–3.762) | 0.008 |  | 1.106 (0.576–2.123) | 0.763 |
| Male sex | 2.366 (1.341–4.174) | 0.003 |  | 1.560 (0.792–3.074) | 0.199 |
| BMI | 1.106 (1.040–1.177) | 0.001 |  |  |  |
| *BMI ≥ 25* | 1.811 (1.070–3.064) | 0.027 |  | 1.555 (0.889–2.721) | 0.122 |
| *BMI ≥ 30* | 1.719 (0.779–3.792) | 0.180 |  |  |  |
| Sx onset to admission, days | 1.082 (0.955–1.225) | 0.216 |  |  |  |
| **Initial presentation** |  |  |  |  |  |
| Initial Ct value (NP swab, RdRp) | 0.999 (0.949–1.051) | 0.961 |  |  |  |
| Pneumonia | 1.342 (0.751–2.398) | 0.320 |  |  |  |
| SIRS | 1.687 (0.935–3.042) | 0.082 |  |  |  |
| SpO_2_ | 0.640 (0.519–0.790) | <0.001 |  |  |  |
| *SpO_2_ <97%* | 3.697 (2.198–6.218) | <0.001 |  | 2.697 (1.545–4.708) | <0.001 |
| **Initial laboratory tests** |  |  |  |  |  |
| WBC count, x10^3^/μL | 1.095 (0.938–1.279) | 0.250 |  |  |  |
| Neutrophil count, x10^3^/μL | 1.204 (1.030–1.406) | 0.020 |  |  |  |
| *Neutrophil > 3500 /μL* | 1.877 (1.101–3.200) | 0.021 |  | 1.283 (0.717–2.297) | 0.402 |
| Lymphocyte count, x10^3^/μL | 0.497 (0.262–0.941) | 0.032 |  |  |  |
| *Lymphopenia (<1500/μL)* | 1.353 (0.797–2.297) | 0.263 |  |  |  |
| Platelet count, x10^3^/μL | 0.988 (0.983–0.993) | <0.001 |  |  |  |
| *Thrombocytopenia (<150 x10^3^/μL)* | 2.745 (1.627–4.631) | <0.001 |  | 1.720 (0.958–3.087) | 0.069 |
| Total bilirubin, mg/dL | 1.052 (0.369–3.003) | 0.924 |  |  |  |
| Albumin, g/dL | 0.369 (0.173–0.789) | 0.010 |  |  |  |
| *Albumin < 4.0 g/dL* | 2.691 (1.422–5.093) | 0.002 |  | 1.574 (0.750–3.303) | 0.230 |
| AST, IU/L | 1.006 (0.996–1.017) | 0.241 |  |  |  |
| ALT, IU/L | 1.003 (0.994–1.013) | 0.503 |  |  |  |
| BUN, mg/dL | 1.064 (1.019–1.111) | 0.005 |  |  |  |
| *BUN elevation (>19 mg/dL)* | 2.227 (1.198–4.138) | 0.011 |  |  |  |
| Creatinine, mg/dL | 5.592 (2.846–10.988) | <0.001 |  | 2.028 (0.681–6.041) | 0.204 |
| *Creatinine elevation (>1.2 mg/dL)* | 3.993 (2.017–7.907) | <0.001 |  |  |  |
| CPK, IU/L | 1.001 (1.000–1.002) | 0.007 |  | 1.001 (1.000–1.002) | 0.199 |
| *CPK elevation (>250 IU/L)* | 2.058 (0.974–4.349) | 0.059 |  |  |  |
| LDH, IU/L | 1.004 (1.002–1.005) | <0.001 |  |  |  |
| *LDH elevation (>400 IU/L)* | 1.868 (1.109–3.146) | 0.019 |  | 0.920 (0.513–1.650) | 0.780 |
| CRP, mg/dL | 1.162 (1.104–1.222) | <0.001 |  |  |  |
| *CRP elevation (>1.5 mg/dL)* | 4.674 (2.742–7.970) | <0.001 |  | 2.414 (1.292–4.509) | 0.006 |
| *CRP elevation (>5 mg/dL)* | 4.448 (2.465–8.027) | <0.001 |  |  |  |
| **Underlying diseases** |  |  |  |  |  |
| Cardiovascular disease | 0.836 (0.333–2.095) | 0.702 |  |  |  |
| Respiratory disease | 0.287 (0.040–2.072) | 0.216 |  |  |  |
| Diabetes mellitus | 0.780 (0.369–1.648) | 0.515 |  |  |  |
| Hypertension | 1.945 (1.157–3.270) | 0.012 |  | 1.403 (0.792–2.486) | 0.246 |
| Liver disease | 0.048 (0.000–539.455) | 0.808 |  |  |  |
| Renal disease | 4.486 (0.620–32.446) | 0.137 |  |  |  |
| Charlson Comorbidity Index | 0.761 (0.475–1.220) | 0.257 |  |  |  |
| **Regdanvimab treatment** | 0.221 (0.088–0.554) | 0.001 |  | 0.262 (0.103–0.667) | 0.005 |

Abbreviations: HR, hazard ratio; CI, confidence interval; BMI, body mass index; Sx, symptom; Ct, cycle threshold; NP, nasopharyngeal; RdRp, RNA-dependent RNA polymerase; SIRS, systemic inflammatory response syndrome; SpO_2_, saturation of percutaneous oxygen; WBC, white blood cell; AST, aspartate aminotransferase; ALT, alanine aminotransferase; BUN, blood urea nitrogen; CPK, creatine phosphokinase; LDH, lactate dehydrogenase; CRP, C-reactive protein

**Supplementary Table 3. Univariate and multivariate analyses of the 21-day probability of progression to O_2_ support via nasal prong (supportive care group, n = 544)**

| **Variables** | ***Univariate analysis*** | |  | ***Multivariate analysis*** | |
| --- | --- | --- | --- | --- | --- |
|  | **HR (95% CI)** | ***P* value** |  | **HR (95% CI)** | ***P* value** |
| **Demographics** |  |  |  |  |  |
| Age, years | 1.039 (1.023–1.055) | <0.001 |  |  |  |
| *Age ≥ 60 years* | 2.273 (1.498–3.449) | <0.001 |  |  |  |
| *Age ≥ 70 years* | 2.861 (1.917–4.269) | <0.001 |  | 1.959 (1.256–3.054) | 0.003 |
| Male sex | 1.545 (1.0.37–2.302) | 0.032 |  | 0.919 (0.569–1.485) | 0.919 |
| BMI | 1.075 (1.022–1.131) | 0.005 |  |  |  |
| *BMI ≥ 25* | 1.500 (1.014–2.221) | 0.043 |  | 1.289 (0.848–1.959) | 0.235 |
| *BMI ≥ 30* | 1.162 (0.564–2.393) | 0.684 |  |  |  |
| Sx onset to admission, days | 1.112 (1.013–1.221) | 0.026 |  |  |  |
| **Initial presentation** |  |  |  |  |  |
| Initial Ct value (NP swab, RdRp) | 0.997 (0.961–1.036) | 0.892 |  |  |  |
| Pneumonia | 1.068 (0.706–1.615) | 0.756 |  |  |  |
| SIRS | 1.500 (0.942–2.390) | 0.088 |  |  |  |
| SpO_2_ | 0.583 (0.500–0.681) | <0.001 |  |  |  |
| *SpO_2_ <97%* | 3.269 (2.207–4.842) | <0.001 |  | 2.389 (1.576–3.619) | <0.001 |
| **Initial laboratory tests** |  |  |  |  |  |
| WBC count, x10^3^/μL | 1.169 (1.048–1.305) | 0.005 |  |  |  |
| Neutrophil count, x10^3^/μL | 1.260 (1.126–1.409) | <0.001 |  |  |  |
| *Neutrophil > 3500 /μL* | 2.283 (1.532–3.401) | <0.001 |  | 1.817 (1.171–2.820) | 0.008 |
| Lymphocyte count, x10^3^/μL | 0.624 (0.399–0.997) | 0.039 |  |  |  |
| *Lymphopenia (<1500/μL)* | 1.244 (0.829–1.868) | 0.291 |  |  |  |
| Platelet count, x10^3^/μL | 0.987 (0.983–0.991) | <0.001 |  |  |  |
| *Thrombocytopenia (<150 x10^3^/μL)* | 2.945 (1.985–4.369) | <0.001 |  | 2.222 (1.435–3.441) | <0.001 |
| Total bilirubin, mg/dL | 0.856 (0.357–2.050) | 0.727 |  |  |  |
| Albumin, g/dL | 0.265 (0.148–0.472) | <0.001 |  |  |  |
| *Albumin < 4.0 g/dL* | 2.700 (1.681–4.335) | <0.001 |  | 1.269 (0.757–2.127) | 0.366 |
| AST, IU/L | 1.004 (0.996–1.013) | 0.311 |  |  |  |
| ALT, IU/L | 1.000 (0.992–1.008) | 0.930 |  |  |  |
| BUN, mg/dL | 1.077 (1.046–1.110) | <0.001 |  |  |  |
| *BUN elevation (>19 mg/dL)* | 2.714 (1.745–4.222) | <0.001 |  |  |  |
| Creatinine, mg/dL | 4.540 (2.756–7.480) | <0.001 |  | 2.200 (1.035–4.675) | 0.040 |
| *Creatinine elevation (>1.2 mg/dL)* | 3.495 (2.073–5.895) | <0.001 |  |  |  |
| CPK, IU/L | 1.001 (1.000–1.002) | 0.004 |  |  |  |
| *CPK elevation (>250 IU/L)* | 2.246 (1.297–3.889) | 0.004 |  | 3.296 (2.080–5.223) | <0.001 |
| LDH, IU/L | 1.003 (1.002–1.005) | <0.001 |  |  |  |
| *LDH elevation (>400 IU/L)* | 2.148 (1.442–3.201) | <0.001 |  | 1.355 (0.873–2.105) | 0.176 |
| CRP, mg/dL | 1.183 (1.138–1.231) | <0.001 |  |  |  |
| *CRP elevation (>1.5 mg/dL)* | 5.597 (3.717–8.430) | <0.001 |  | 3.296 (2.080–5.223) | <0.001 |
| *CRP elevation (>5 mg/dL)* | 4.058 (2.576–6.391) | <0.001 |  |  |  |
| **Underlying diseases** |  |  |  |  |  |
| Cardiovascular disease | 1.753 (1.012–3.035) | 0.045 |  |  |  |
| Respiratory disease | 1.361 (0.661–2.804) | 0.403 |  |  |  |
| Diabetes mellitus | 1.263 (0.774–2.061) | 0.351 |  |  |  |
| Hypertension | 2.129 (1.439–3.152) | <0.001 |  | 1.589 (1.027–2.459) | 0.038 |
| Liver disease | 1.619 (0.399–6.564) | 0.500 |  |  |  |
| Renal disease | 2.682 (0.373–19.265) | 0.327 |  |  |  |
| Charlson Comorbidity Index | 1.409 (1.094–1.816) | 0.008 |  |  |  |

Abbreviations: HR, hazard ratio; CI, confidence interval; BMI, body mass index; Sx, symptom; Ct, cycle threshold; NP, nasopharyngeal; RdRp, RNA-dependent RNA polymerase; SIRS, systemic inflammatory response syndrome; SpO_2_, saturation of percutaneous oxygen; WBC, white blood cell; AST, aspartate aminotransferase; ALT, alanine aminotransferase; BUN, blood urea nitrogen; CPK, creatine phosphokinase; LDH, lactate dehydrogenase; CRP, C-reactive protein

**Supplementary Table 4. Univariate and multivariate analyses of the 21-day probability of progression to severe disease (supportive care group, n = 544)**

| **Variables** | ***Univariate analysis*** | |  | ***Multivariate analysis*** | |
| --- | --- | --- | --- | --- | --- |
|  | **HR (95% CI)** | ***P* value** |  | **HR (95% CI)** | ***P* value** |
| **Demographics** |  |  |  |  |  |
| Age, years | 1.016 (0.997–1.036) | 0.099 |  |  |  |
| *Age ≥ 60 years* | 1.170 (0.677–2.024) | 0.574 |  |  |  |
| *Age ≥ 70 years* | 1.805 (1.007–3.235) | 0.047 |  | 1.205 (0.615–2.358) | 0.587 |
| Male sex | 2.257 (1.264–4.030) | 0.006 |  | 1.411 (0.704–2.829) | 0.332 |
| BMI | 1.142 (1.072–1.217) | <0.001 |  |  |  |
| *BMI ≥ 25* | 2.296 (1.313–4.015) | 0.004 |  | 1.860 (1.029–3.362) | 0.040 |
| *BMI ≥ 30* | 2.171 (0.979–4.816) | 0.056 |  |  |  |
| Sx onset to admission, days | 1.030 (0.908–1.169) | 0.642 |  |  |  |
| **Initial presentation** |  |  |  |  |  |
| Initial Ct value (NP swab, RdRp) | 0.998 (0.943–1.056) | 0.950 |  |  |  |
| Pneumonia | 1.285 (0.712–2.319) | 0.405 |  |  |  |
| SIRS | 1.324 (0.680–2.575) | 0.409 |  |  |  |
| SpO_2_ | 0.655 (0.531–0.806) | <0.001 |  |  |  |
| *SpO_2_ <97%* | 3.506 (2.034–6.041) | <0.001 |  | 2.439 (1.357–4.383) | 0.003 |
| **Initial laboratory tests** |  |  |  |  |  |
| WBC count, x10^3^/μL | 1.117 (0.950–1.313) | 0.180 |  |  |  |
| Neutrophil count, x10^3^/μL | 1.233 (1.048–1.452) | 0.012 |  |  |  |
| *Neutrophil > 3500 /μL* | 2.094 (1.203–3.644) | 0.009 |  | 1.407 (0.764–2.591) | 0.273 |
| Lymphocyte count, x10^3^/μL | 0.482 (0.248–0.935) | 0.031 |  |  |  |
| *Lymphopenia (<1500/μL)* | 1.568 (0.905–2.719) | 0.109 |  |  |  |
| Platelet count, x10^3^/μL | 0.989 (0.984–0.995) | <0.001 |  |  |  |
| *Thrombocytopenia (<150 x10^3^/μL)* | 2.487 (1.436–4.307) | 0.001 |  | 1.487 (0.806–2.744) | 0.204 |
| Total bilirubin, mg/dL | 1.376 (0.432–4.382) | 0.590 |  |  |  |
| Albumin, g/dL | 0.462 (0.209–1.020) | 0.056 |  |  |  |
| *Albumin < 4.0 g/dL* | 2.217 (1.139–4.316) | 0.019 |  | 1.368 (0.629–2.978) | 0.429 |
| AST, IU/L | 1.007 (0.997–1.017) | 0.188 |  |  |  |
| ALT, IU/L | 1.005 (0.996–1.014) | 0.305 |  |  |  |
| BUN, mg/dL | 1.061 (1.016–1.108) | 0.007 |  |  |  |
| *BUN elevation (>19 mg/dL)* | 2.216 (1.182–4.154) | 0.013 |  |  |  |
| Creatinine, mg/dL | 4.678 (2.371–9.231) | <0.001 |  | 2.184 (0.725–6.579) | 0.165 |
| *Creatinine elevation (>1.2 mg/dL)* | 3.809 (1.910–7.595) | <0.001 |  |  |  |
| CPK, IU/L | 1.001 (1.001–1.002) | 0.002 |  |  |  |
| *CPK elevation (>250 IU/L)* | 2.188 (1.030–4.649) | 0.042 |  | 1.116 (0.497–2.507) | 0.790 |
| LDH, IU/L | 1.004 (1.002–1.006) | <0.001 |  |  |  |
| *LDH elevation (>400 IU/L)* | 1.803 (1.043–3.116) | 0.035 |  | 0.954 (0.517–1.761) | 0.881 |
| CRP, mg/dL | 1.138 (1.080–1.200) | <0.001 |  |  |  |
| *CRP elevation (>1.5 mg/dL)* | 4.725 (2.686–8.313) | <0.001 |  | 2.711 (1.411–5.209) | 0.003 |
| *CRP elevation (>5 mg/dL)* | 3.941 (2.133–7.280) | <0.001 |  |  |  |
| **Underlying diseases** |  |  |  |  |  |
| Cardiovascular disease | 0.919 (0.365–2.311) | 0.857 |  |  |  |
| Respiratory disease | 0.268 (0.037–1.940) | 0.192 |  |  |  |
| Diabetes mellitus | 0.755 (0.341–1.675) | 0.490 |  |  |  |
| Hypertension | 2.050 (1.190–3.533) | 0.010 |  | 1.357 (0.744–2.474) | 1.357 |
| Liver disease | 0.048 (0.000–1200.561) | 0.558 |  |  |  |
| Renal disease | 5.301 (0.730–38.473) | 0.099 |  |  |  |
| Charlson Comorbidity Index | 0.741 (0.457–1.203) | 0.226 |  |  |  |

Abbreviations: HR, hazard ratio; CI, confidence interval; BMI, body mass index; Sx, symptom; Ct, cycle threshold; NP, nasopharyngeal; RdRp, RNA-dependent RNA polymerase; SIRS, systemic inflammatory response syndrome; SpO_2_, saturation of percutaneous oxygen; WBC, white blood cell; AST, aspartate aminotransferase; ALT, alanine aminotransferase; BUN, blood urea nitrogen; CPK, creatine phosphokinase; LDH, lactate dehydrogenase; CRP, C-reactive protein

**Supplementary Table 5. Univariate and multivariate analyses for 21-day** **probability of progression to severe disease (high risk group with additional risk factors, n = 319)**

| **Variables** | ***Univariate analysis*** | |  | ***Multivariate analysis*** | |
| --- | --- | --- | --- | --- | --- |
|  | **HR (95% CI)** | ***P* value** |  | **HR (95% CI)** | ***P* value** |
| **Demographics** |  |  |  |  |  |
| Age, years | 1.020 (0.997–1.044) | 0.087 |  |  |  |
| *Age ≥ 60 years* | 1.367 (0.758–2.465) | 0.299 |  |  |  |
| *Age ≥ 70 years* | 1.906 (1.026–3.538) | 0.041 |  | 1.169 (0.605–2.257) | 0.642 |
| Male sex | 1.892 (0.995–3.596) | 0.052 |  |  |  |
| BMI | 1.113 (1.039–1.192) | 0.002 |  | 1.085 (1.008–1.168) | 0.030 |
| *BMI ≥ 25* | 1.580 (0.669–3.732) | 0.297 |  |  |  |
| *BMI ≥ 30* | 0.944 (0.457–1.951) | 0.877 |  |  |  |
| Sx onset to admission, days | 1.049 (0.914–1.204) | 0.497 |  |  |  |
| **Initial presentations** |  |  |  |  |  |
| Initial Ct value (NP swab, RdRp) | 0.967 (0.908–1.029) | 0.286 |  |  |  |
| Pneumonia | 1.103 (0.587–2.071) | 0.761 |  |  |  |
| SIRS | 1.477 (0.777–2.806) | 0.234 |  |  |  |
| SpO_2_ | 0.900 (0.701–1.154) | 0.405 |  |  |  |
| *SpO_2_ <97%* | 1.350 (0.736–2.476) | 0.333 |  |  |  |
| **Initial laboratory tests** |  |  |  |  |  |
| WBC count, x10^3^/μL | 0.975 (0.818–1.162) | 0.777 |  |  |  |
| Neutrophil count, x10^3^/μL | 1.036 (0.860–1.249) | 0.710 |  |  |  |
| *Neutrophil > 3500 /μL* | 1.131 (0.621–2.058) | 0.687 |  |  |  |
| Lymphocyte count, x10^3^/μL | 0.623 (0.315–1.232) | 0.174 |  |  |  |
| *Lymphopenia (<1500/μL)* | 1.085 (0.607–1.940) | 0.782 |  |  |  |
| Platelet count, x10^3^/μL | 0.990 (0.985–0.996) | 0.001 |  |  |  |
| *Thrombocytopenia (<150 x10^3^/μL)* | 2.761 (1.542–4.943) | 0.001 |  | 2.079 (1.117–3.870) | 0.021 |
| Total bilirubin, mg/dL | 1.508 (0.395–5.761) | 0.548 |  |  |  |
| Albumin, g/dL | 0.655 (0.287–1.494) | 0.315 |  |  |  |
| *Albumin < 4.0 g/dL* | 1.812 (0.919–3.572) | 0.086 |  |  |  |
| AST, IU/L | 1.005 (0.992–1.017) | 0.459 |  |  |  |
| ALT, IU/L | 1.004 (0.993–1.015) | 0.488 |  |  |  |
| BUN, mg/dL | 1.032 (0.982–1.084) | 0.212 |  |  |  |
| *BUN elevation (>19 mg/dL)* | 1.381 (0.685–2.784) | 0.367 |  |  |  |
| Creatinine, mg/dL | 4.898 (1.929–12.435) | 0.001 |  | 1.702 (0.616–4.701) | 0.305 |
| *Creatinine elevation (>1.2 mg/dL)* | 3.134 (1.512–6.497) | 0.002 |  |  |  |
| CPK, IU/L | 1.001 (1.000–1.001) | 0.220 |  |  |  |
| *CPK elevation (>250 IU/L)* | 1.084 (0.459–2.557) | 0.854 |  |  |  |
| LDH, IU/L | 1.002 (1.000–1.004) | 0.026 |  | 1.001 (0.999–1.003) | 0.323 |
| *LDH elevation (>400 IU/L)* | 1.263 (0.698–2.283) | 0.440 |  |  |  |
| CRP, mg/dL | 1.077 (1.009–1.150) | 0.026 |  |  |  |
| *CRP elevation (>1.5 mg/dL)* | 1.798 (0.913–3.540) | 0.090 |  |  |  |
| *CRP elevation (>5 mg/dL)* | 2.104 (1.134–3.903) | 0.018 |  | 1.481 (0.728–3.015) | 0.278 |
| **Underlying diseases** |  |  |  |  |  |
| Cardiovascular diseases | 1.063 (0.420–2.691) | 0.898 |  |  |  |
| Respiratory diseases | 0.281 (0.039–2.039) | 0.209 |  |  |  |
| Diabetes mellitus | 0.722 (0.323–1.615) | 0.428 |  |  |  |
| Hypertension | 2.490 (1.390–4.461) | 0.002 |  | 1.983 (1.068–3.681) | 0.030 |
| Liver diseases | 0.048 (0.000–870.346) | 0.545 |  |  |  |
| Renal diseases | 4.010 (0.552–29.140) | 0.170 |  |  |  |
| Charlson Comorbidity Index | 0.710 (0.428–1.178) | 0.185 |  |  |  |
| **Regdanvimab treatment** | 0.171 (0.053–0.551) | 0.003 |  | 0.202 (0.062–0.657) | 0.008 |

Abbreviations: HR, hazard ratio; CI, confidence interval; BMI, body mass index; Sx, symptom; Ct, cycle threshold; NP, nasopharyngeal; RdRp, RNA-dependent RNA polymerase; SIRS, systemic inflammatory response syndrome; SpO_2_, saturation of percutaneous oxygen; WBC, white blood cell; AST, aspartate aminotransferase; ALT, alanine aminotransferase; BUN, blood urea nitrogen; CPK, creatine phosphokinase; LDH, lactate dehydrogenase; CRP, C-reactive protein

**Supplementary Table 6. Univariate and multivariate analyses of the 21-day probability of progression to O_2_ support via nasal prong (regdanvimab group, n = 234)**

| **Variables** | ***Univariate analysis*** | |  | ***Multivariate analysis*** | |
| --- | --- | --- | --- | --- | --- |
|  | **HR (95% CI)** | ***P* value** |  | **HR (95% CI)** | ***P* value** |
| **Demographics** |  |  |  |  |  |
| Age, years | 1.058 (1.018–1.100) | 0.004 |  |  |  |
| *Age ≥ 60 years* | 4.214 (1.602–11.090) | 0.004 |  | 3.366 (1.123–10.091) | 0.030 |
| *Age ≥ 70 years* | 4.013 (1.445–11.149) | 0.008 |  |  |  |
| Male sex | 1.397 (0.550–3.547) | 0.482 |  |  |  |
| BMI | 0.965 (0.845–1.103) | 0.604 |  |  |  |
| *BMI ≥ 25* | 0.996 (0.405–2.451) | 0.993 |  |  |  |
| *BMI ≥ 30* | 0.528 (0.070–3.953) | 0.534 |  |  |  |
| Sx onset to admission, days | 1.492 (1.151–1.933) | 0.003 |  | 1.512 (1.079–2.119 | 0.016 |
| Sx onset to regdanvimab Tx | 1.327 (1.006–1.750) | 0.045 |  |  |  |
| **Initial presentation** |  |  |  |  |  |
| Initial Ct value (NP swab, RdRp) | 1.018 (0.952–1.090) | 0.595 |  |  |  |
| Pneumonia | 0.676 (0.266–1.717) | 0.410 |  |  |  |
| SIRS | 1.861 (0.707–4.896) | 0.208 |  |  |  |
| SpO_2_ | 0.531 (0.338–0.833) | 0.006 |  |  |  |
| *SpO_2_ <97%* | 4.174 (1.696–10.275) | 0.002 |  | 4.754 (1.612–14.022) | 0.005 |
| **Initial laboratory tests** |  |  |  |  |  |
| WBC count, x10^3^/μL | 0.798 (0.549–1.160) | 0.237 |  |  |  |
| Neutrophil count, x10^3^/μL | 0.996 (0.952–1.042) | 0.868 |  |  |  |
| *Neutrophil > 3500 /μL* | 0.527 (0.153–1.808) | 0.308 |  |  |  |
| Lymphocyte count, x10^3^/μL | 0.566 (0.185–1.728) | 0.318 |  |  |  |
| *Lymphopenia (<1500/μL)* | 1.987 (0.807–4.889) | 0.135 |  |  |  |
| Platelet count, x10^3^/μL | 0.981 (0.971–0.990) | <0.001 |  |  |  |
| *Thrombocytopenia (<150 x10^3^/μL)* | 5.604 (2.253–13.938) | <0.001 |  | 3.605 (1.319–9.849) | 0.012 |
| Total bilirubin, mg/dL | 0.438 (0.067–2.859) | 0.388 |  |  |  |
| Albumin, g/dL | 0.265 (0.148–0.472) | <0.001 |  |  |  |
| *Albumin < 4.0 g/dL* | 5.657 (1.876–17.059) | 0.002 |  | 1.255 (0.349–4.505) | 1.255 |
| AST, IU/L | 1.006 (0.987–1.025) | 0.534 |  |  |  |
| ALT, IU/L | 1.000 (0.982–1.019) | 0.959 |  |  |  |
| BUN, mg/dL | 1.079 (0.981–1.187) | 0.118 |  |  |  |
| *BUN elevation (>19 mg/dL)* | 2.197 (0.640–7.540) | 0.211 |  |  |  |
| Creatinine, mg/dL | 8.236 (0.871–77.923) | 0.066 |  |  |  |
| *Creatinine elevation (>1.2 mg/dL)* | 1.967 (0.263–14.739) | 0.510 |  |  |  |
| CPK, IU/L | 0.999 (0.996–1.003) | 0.751 |  |  |  |
| *CPK elevation (>250 IU/L)* | 1.820 (0.420–7.877) | 0.423 |  |  |  |
| LDH, IU/L | 1.005 (1.002–1.008) | <0.001 |  |  |  |
| *LDH elevation (>400 IU/L)* | 2.829 (1.138–7.033) | 0.025 |  | 1.901 (0.713–5.065) | 0.199 |
| CRP, mg/dL | 1.296 (1.115–1.506) | 0.001 |  |  |  |
| *CRP elevation (>1.5 mg/dL)* | 3.308 (1.344–8.143) | 0.009 |  | 1.704 (0.415–6.993) | 0.460 |
| *CRP elevation (>5 mg/dL)* | 3.910 (1.139–13.424) | 0.030 |  |  |  |
| **Underlying diseases** |  |  |  |  |  |
| Cardiovascular disease | 3.574 (1.287–9.926) | 0.015 |  | 2.995 (0.905–9.909) | 0.072 |
| Respiratory disease | 1.615 (0.216–12.095) | 0.641 |  |  |  |
| Diabetes mellitus | 2.352 (0.894–6.188) | 0.083 |  |  |  |
| Hypertension | 2.256 (0.917–5.553) | 0.077 |  |  |  |
| Liver disease | 0.048 (0.000–198506.333) | 0.697 |  |  |  |
| Renal disease | 0.049 (0.000–7.333E+11) | 0.846 |  |  |  |
| Charlson Comorbidity Index | 1.689 (0.936–3.048) | 0.082 |  |  |  |

Abbreviations: HR, hazard ratio; CI, confidence interval; BMI, body mass index; Sx, symptom; Ct, cycle threshold; NP, nasopharyngeal; RdRp, RNA-dependent RNA polymerase; SIRS, systemic inflammatory response syndrome; SpO_2_, saturation of percutaneous oxygen; WBC, white blood cell; AST, aspartate aminotransferase; ALT, alanine aminotransferase; BUN, blood urea nitrogen; CPK, creatine phosphokinase; LDH, lactate dehydrogenase; CRP, C-reactive protein

**Supplementary Table 7. Univariate and multivariate analyses of the 21-day probability of progression to severe disease (regdanvimab group, n = 234)**

| **Variables** | ***Univariate analysis*** | |  | ***Multivariate analysis*** | |
| --- | --- | --- | --- | --- | --- |
|  | **HR (95% CI)** | ***P* value** |  | **HR (95% CI)** | ***P* value** |
| **Demographics** |  |  |  |  |  |
| Age, years | 1.033 (0.965–1.107) | 0.350 |  |  |  |
| *Age ≥ 60 years* | 7.215 (0.805–64.658) | 0.077 |  |  |  |
| *Age ≥ 70 years* | 2.309 (0.257–20.768) | 0.455 |  |  |  |
| Male sex | 54.181 (0.039–76062.048) | 0.280 |  |  |  |
| BMI | 0.801 (0.589–1.090) | 0.158 |  |  |  |
| *BMI ≥ 25* | 0.274 (0.031–2.455) | 0.247 |  |  |  |
| *BMI ≥ 30* | 0.043 (0.000–19787.024) | 0.636 |  |  |  |
| Sx onset to admission, days | 1.270 (0.793–2.034) | 0.320 |  |  |  |
| Sx onset to regdanvimab Tx | 0.842 (0.524–1.352) | 0.476 |  |  |  |
| **Initial presentation** |  |  |  |  |  |
| Initial Ct value (NP swab, RdRp) | 0.971 (0.840–1.123) | 0.692 |  |  |  |
| Pneumonia | 0.34.6242 (0.010–119914.932) | 0.394 |  |  |  |
| SIRS | 17.087 (1.907–153.079) | 0.011 |  | 20.449 (1.986–210.503) | 0.011 |
| SpO_2_ | 0.661 (0.285–1.535) | 0.336 |  |  |  |
| *SpO_2_ <97%* | 5.360 (0.895–32.083) | 0.066 |  |  |  |
| **Initial laboratory tests** |  |  |  |  |  |
| WBC count, x10^3^/μL | 0.786 (0.376–1.640) | 0.520 |  |  |  |
| Neutrophil count, x10^3^/μL | 0.840 (0.376–1.877) | 0.671 |  |  |  |
| *Neutrophil > 3500 /μL* | 0.686 (0.077–6.138) | 0.736 |  |  |  |
| Lymphocyte count, x10^3^/μL | 0.473 (0.049–4.533) | 0.516 |  |  |  |
| *Lymphopenia (<1500/μL)* | 0.429 (0.048–3.837) | 0.449 |  |  |  |
| Platelet count, x10^3^/μL | 0.977 (0.957–0.996) | 0.021 |  | 0.982 (0.960–1.004) | 0.104 |
| *Thrombocytopenia (<150 x10^3^/μL)* | 5.343 (0.891–32.048) | 0.067 |  |  |  |
| Total bilirubin, mg/dL | 0.499 (0.014–17.324) | 0.701 |  |  |  |
| Albumin, g/dL | 0.202 (0.014–2.868) | 0.237 |  |  |  |
| *Albumin < 4.0 g/dL* | 4.752 (0.530–42.614) | 0.164 |  |  |  |
| AST, IU/L | 0.988 (0.923–1.058) | 0.737 |  |  |  |
| ALT, IU/L | 0.974 (0.908–1.045) | 0.465 |  |  |  |
| BUN, mg/dL | 0.896 (0.685–1.172) | 0.423 |  |  |  |
| *BUN elevation (>19 mg/dL)* | 0.043 (0.000–45091.651) | 0.657 |  |  |  |
| Creatinine, mg/dL | 12.005 (0.171–844.886) | 0.252 |  |  |  |
| *Creatinine elevation (>1.2 mg/dL)* | 0.047 (0.000–248073309.2) | 0.789 |  |  |  |
| CPK, IU/L | 0.999 (0.991–1.007) | 0.788 |  |  |  |
| *CPK elevation (>250 IU/L)* | 0.045 (0.000–349643.877) | 0.702 |  |  |  |
| LDH, IU/L | 1.004 (0.998–1.009) | 0.167 |  |  |  |
| *LDH elevation (>400 IU/L)* | 1.310 (0.219–7.839) | 0.769 |  |  |  |
| CRP, mg/dL | 1.350 (1.030–1.768) | 0.030 |  | 1.374 (0.880–2.145) | 0.162 |
| *CRP elevation (>1.5 mg/dL)* | 2.388 (0.398–14.312) | 0.341 |  |  |  |
| *CRP elevation (>5 mg/dL)* | 4.818 (0.538–43.118) | 0.160 |  |  |  |
| **Underlying diseases** |  |  |  |  |  |
| Cardiovascular disease | 0.042 (0.000–15734.958) | 0.629 |  |  |  |
| Respiratory disease | 0.047 (0.000–40597799.60) | 0.771 |  |  |  |
| Diabetes mellitus | 1.174 (0.131–10.512) | 0.886 |  |  |  |
| Hypertension | 1.310 (0.219–7.839) | 0.768 |  |  |  |
| Liver disease | 0.048 (0.000–1.769E+11) | 0.837 |  |  |  |
| Renal disease | 0.049 (0.000–5.135E+23) | 0.919 |  |  |  |
| Charlson Comorbidity Index | 0.635 (0.091–4.415) | 0.647 |  |  |  |

Abbreviations: HR, hazard ratio; CI, confidence interval; BMI, body mass index; Sx, symptom; Ct, cycle threshold; NP, nasopharyngeal; RdRp, RNA-dependent RNA polymerase; SIRS, systemic inflammatory response syndrome; SpO_2_, saturation of percutaneous oxygen; WBC, white blood cell; AST, aspartate aminotransferase; ALT, alanine aminotransferase; BUN, blood urea nitrogen; CPK, creatine phosphokinase; LDH, lactate dehydrogenase; CRP, C-reactive protein

**Supplementary Table 8. Baseline characteristics of the regdanvimab and supportive care groups in the PS-matched cohorts (n = 468)**

| **Variables** | **Regdanvimab**  **(n =234)** | **Supportive care**  **(n = 234)** | ***P* value** |
| --- | --- | --- | --- |
| **Demographics** |  |  |  |
| Age, years | 51.8 ± 14.3 | 51.7 ± 15.7 | 0.975 |
| *Age ≥ 60 years* | 81 (34.6) | 85 (36.3) | 0.772 |
| *Age ≥ 70 years* | 21 (9.0) | 28 (12.0) | 0.365 |
| Male sex | 130 (55.6) | 133 (56.8) | 0.852 |
| BMI | 25.2 ± 3.6 | 24.9 ± 3.7 | 0.372 |
| *BMI ≥ 25* | 111 (47.4) | 103 (44.0) | 0.516 |
| *BMI ≥ 30* | 22 (9.4) | 18 (7.7) | 0.620 |
| Sx onset to admission, days | 2.8 ± 2.0 | 2.8 ± 2.1 | 0.928 |
| **Initial presentation** |  |  |  |
| Initial Ct value (NP swab, RdRp) | 19.2 ± 6.4 | 19.9 ± 5.8 | 0.302 |
| Pneumonia | 168 (71.8) | 161 (68.8) | 0.544 |
| SIRS | 47 (20.1) | 35 (15.0) | 0.181 |
| SpO_2_ | 97.2 ± 1.0 | 97.3 ± 1.2 | 0.368 |
| *SpO_2_ < 97%* | 52 (22.2) | 53 (22.6) | 1.000 |
| **Initial laboratory tests** |  |  |  |
| WBC count, /μL | 4749.4 ± 1505.4 | 4728.8 ± 1456.0 | 0.880 |
| *Leukopenia (<4000 /μL)* | 73 (31.2) | 72 (30.8) | 1.000 |
| Lymphocyte count, /μL | 1202.1 ± 460.9 | 1209.4 ± 473.3 | 0.866 |
| *Lymphopenia (<1500/μL)* | 85 (36.3) | 83 (35.5) | 0.923 |
| Platelet count, x10^3^/μL | 199.7 ± 61.3 | 193.1 ± 60.0 | 0.241 |
| *Thrombocytopenia (<150 x10^3^/μL)* | 49 (20.9) | 54 (24.1) | 0.655 |
| Total bilirubin, mg/dL | 0.6 ± 0.3 | 0.6 ± 0.2 | 0.442 |
| Albumin, g/dL | 4.4 ± 0.3 | 4.4 ± 0.3 | 0.812 |
| AST, IU/L | 32.7 ± 18.5 | 32.2 ± 18.3 | 0.740 |
| ALT, IU/L | 33.7 ± 23.6 | 33.8 ± 26.7 | 0.946 |
| BUN, mg/dL | 13.5 ± 4.0 | 13.8 ± 4.2 | 0.354 |
| *BUN elevation (>19 mg/dL)* | 19 (8.1) | 17 (7.3) | 0.862 |
| Creatinine, mg/dL | 0.8 ± 0.2 | 0.9 ± 0.2 | 0.472 |
| *Creatinine elevation (>1.2 mg/dL)* | 7 (3.0) | 11 (4.7) | 0.471 |
| CPK, IU/L | 128.3 ± 185.7 | 125.6 ± 149.1 | 0.861 |
| *CPK elevation (>250 IU/L)* | 15 (6.4) | 17 (7.3) | 0.855 |
| LDH, IU/L | 388.7 ± 100.9 | 386.3 ± 91.7 | 0.788 |
| *LDH elevation (>400 IU/L)* | 79 (33.8) | 91 (38.9) | 0.290 |
| CRP, mg/dL | 1.2 ± 1.7 | 0.9 ± 1.4 | 0.113 |
| *CRP elevation (>1.5 mg/dL)* | 53 (22.6) | 48 (20.5) | 0.653 |
| *CRP elevation (>5 mg/dL)* | 12 (5.1) | 6 (2.6) | 0.229 |
| **Underlying diseases** |  |  |  |
| Cardiovascular disease | 22 (9.4) | 19 (8.1) | 0.744 |
| Respiratory disease | 8 (3.4) | 14 (6.0) | 0.275 |
| Diabetes mellitus | 40 (17.1) | 44 (18.8) | 0.718 |
| Hypertension | 79 (33.8) | 71 (30.3) | 0.488 |
| Liver disease | 4 (1.7) | 5 (2.1) | 1.000 |
| Renal disease | 1 (0.4) | 1 (0.4) | 1.000 |
| Charlson Comorbidity Index | 0 (0–1) | 0 (0-1) | 0.770 |

Data are expressed as the number (%) of patients, mean ± SD, or median (IQR) unless indicated otherwise.

Abbreviations: BMI, body mass index; Sx, symptom; Ct, cycle threshold; NP, nasopharyngeal; RdRp, RNA-dependent RNA polymerase; SIRS, systemic inflammatory response syndrome; SpO_2_, saturation of percutaneous oxygen; WBC, white blood cell; AST, aspartate aminotransferase; ALT, alanine aminotransferase; BUN, blood urea nitrogen; CPK, creatine phosphokinase; LDH, lactate dehydrogenase; CRP, C-reactive protein

**Supplementary Table 9. Univariate and multivariate analyses of the 21-day probability of progression to O_2_ support via nasal prong (PS-matched cohorts, n = 468))**

| **Variables** | ***Univariate analysis*** | |  | ***Multivariate analysis*** | |
| --- | --- | --- | --- | --- | --- |
|  | **HR (95% CI)** | ***P* value** |  | **HR (95% CI)** | ***P* value** |
| **Demographics** |  |  |  |  |  |
| Age, years | 1.045 (1.001–1.004) | <0.001 |  |  |  |
| *Age ≥ 60 years* | 2.853 (1.620–5.024) | <0.001 |  |  |  |
| *Age ≥ 70 years* | 3.297 (2.275–5.027) | 0.006 |  | 0.904 (0.417–1.960) | 0.799 |
| Male sex | 1.189 (0.675–2.093) | 0.550 |  |  |  |
| BMI | 1.021 (0.947–1.100) | 0.590 |  |  |  |
| *BMI ≥ 25* | 1.307 (0.751–2.277) | 0.344 |  |  |  |
| *BMI ≥ 30* | 0.681 (0.212–2.188) | 0.519 |  |  |  |
| Sx onset to admission, days | 1.340 (1.153–1.556) | <0.001 |  | 1.233 (1.052–1.445) | 0.010 |
| **Initial presentation** |  |  |  |  |  |
| Initial Ct value (NP swab, RdRp) | 1.015 (0.968–1.064) | 0.533 |  |  |  |
| Pneumonia | 0.995 (0.543–1.821) | 0.986 |  |  |  |
| SIRS | 1.905 (1.027–3.532) | 0.041 |  | 1.699 (0.885–3.262) | 0.111 |
| SpO_2_ | 0.530 (0.421–0.668) | <0.001 |  |  |  |
| *SpO_2_ <97%* | 4.561 (2.614–7.956) | <0.001 |  | 3.847 (2.111–7.011) | <0.001 |
| **Initial laboratory tests** |  |  |  |  |  |
| WBC count, x10^3^/μL | 1.000 (1.000–1.000) | 0.567 |  |  |  |
| *WBC count > 5500 /μL* | 0.850 (0.425-1.699) | 0.645 |  |  |  |
| *Leukopenia (<4000 /μL)* | 1.043 (0.576–1.889) | 0.890 |  |  |  |
| Neutrophil count, x10^3^/μL | 1.000 (1.000–1.000) | 0.985 |  |  |  |
| *Neutrophil > 3500 /μL* | 1.313 (0.708–2.434) | 0.388 |  |  |  |
| Lymphocyte count, x10^3^/μL | 1.000 (0.999–1.000) | 0.310 |  |  |  |
| *Lymphopenia (<1500/μL)* | 1.320 (0.753–2.315) | 0.332 |  |  |  |
| Platelet count, x10^3^/μL | 0.984 (0.978–0.990) | <0.001 |  |  |  |
| *Thrombocytopenia (<150 x10^3^/μL)* | 2.908 (2.244–6.804) | <0.001 |  | 2.864 (1.563–5.250) | <0.001 |
| Total bilirubin, mg/dL | 0.670 (0.211–2.129) | 0.498 |  |  |  |
| Albumin, g/dL | 0.202 (0.082–0.495) | <0.001 |  |  |  |
| *Albumin < 4.0 g/dL* | 3.318 (1.492–7.380) | <0.003 |  | 1.875 (0.752–4.679) | 0.178 |
| AST, IU/L | 1.006 (0.994–1.019) | 0.299 |  |  |  |
| ALT, IU/L | 1.000 (0.989–1.011) | 0.991 |  |  |  |
| BUN, mg/dL | 1.057 (1.001–1.117) | 0.045 |  |  |  |
| *BUN elevation (>19 mg/dL)* | 2.438 (1.145–5.193) | 0.021 |  | 1.490 (0.628–3.535) | 0.366 |
| Creatinine, mg/dL | 1.910 (0.637–5.732) | 0.248 |  |  |  |
| *Creatinine elevation (>1.2 mg/dL)* | 1.731 (0.539–5.563) | 0.357 |  |  |  |
| CPK, IU/L | 1.000 (0.999–1.002) | 0.870 |  |  |  |
| *CPK elevation (>250 IU/L)* | 1.584 (0.629–3.991) | 0.329 |  |  |  |
| LDH, IU/L | 1.005 (1.003–1.007) | <0.001 |  |  |  |
| *LDH elevation (>400 IU/L)* | 3.131 (1.769–5.544) | <0.001 |  | 1.813 (0.984–3.339) | 0.056 |
| CRP, mg/dL | 1.353 (1.236–1.481) | <0.001 |  |  |  |
| *CRP elevation (>1.5 mg/dL)* | 5.487 (3.137–9.597) | <0.001 |  |  |  |
| *CRP elevation (>5 mg/dL)* | 6.514 (3.052–13.902) | <0.001 |  | 3.613 (1.569–8.319) | 0.003 |
| **Underlying diseases** |  |  |  |  |  |
| Cardiovascular disease | 3.213 (1.645–6.276) | 0.001 |  | 3.456 (1.617–7.385) | 0.001 |
| Respiratory disease | 1.320 (0.411–4.241) | 0.641 |  |  |  |
| Diabetes mellitus | 1.861 (1.004–3.451) | 0.049 |  | 1.684 (0.874–3.243) | 0.119 |
| Hypertension | 1.913 (1.097–3.336) | 0.022 |  | 1.546 (0.853–2.802) | 0.151 |
| Liver disease | 1.064 (0.147–7.709) | 0.951 |  |  |  |
| Charlson Comorbidity Index | 1.636 (1.414–2.345) | 0.007 |  |  |  |
| **Regdanvimab treatment** | 0.606 (0.343–1.074) | 0.086 |  | 0.548 (0.301–0.999) | 0.050 |

Abbreviations: HR, hazard ratio; CI, confidence interval; BMI, body mass index; Sx, symptom; Ct, cycle threshold; NP, nasopharyngeal; RdRp, RNA-dependent RNA polymerase; SIRS, systemic inflammatory response syndrome; SpO_2_, saturation of percutaneous oxygen; WBC, white blood cell; AST, aspartate aminotransferase; ALT, alanine aminotransferase; BUN, blood urea nitrogen; CPK, creatine phosphokinase; LDH, lactate dehydrogenase; CRP, C-reactive protein

**Supplementary Table 10. Univariate and multivariate analyses of the 21-day probability of progression to severe disease (PS-matched cohorts, n = 468)**

| **Variables** | ***Univariate analysis*** | |  | ***Multivariate analysis*** | |
| --- | --- | --- | --- | --- | --- |
|  | **HR (95% CI)** | ***P* value** |  | **HR (95% CI)** | ***P* value** |
| **Demographics** |  |  |  |  |  |
| Age, years | 1.023 (0.993–1.054) | 0.130 |  |  |  |
| *Age ≥ 60 years* | 1.720 (0.759–3.900) | 0.194 |  |  |  |
| *Age ≥ 70 years* | 1.887 (0.642–5.547) | 0.249 |  |  |  |
| Male sex | 2.250 (0.887–5.706) | 0.088 |  |  |  |
| BMI | 1.066 (0.961–1.181) | 0.228 |  |  |  |
| *BMI ≥ 25* | 1.568 (0.687–3.575) | 0.285 |  |  |  |
| *BMI ≥ 30* | 1.016 (0.238–4.333) | 0.983 |  |  |  |
| Sx onset to admission, days | 1.145 (0.927–1.414) | 0.208 |  |  |  |
| **Initial presentation** |  |  |  |  |  |
| Initial Ct value (NP swab, RdRp) | 1.038 (0.965–1.117) | 0.314 |  |  |  |
| Pneumonia | 2.854 (0.848–9.604) | 0.090 |  |  |  |
| SIRS | 2.619 (1.110–6.178) | 0.028 |  |  |  |
| SpO_2_ | 0.618 (0.438–0.871) | 0.006 |  |  |  |
| *SpO_2_ <97%* | 4.221 (1.862–9.571) | 0.001 |  | 2.829 (1.186–6.747) | 0.019 |
| **Initial laboratory tests** |  |  |  |  |  |
| WBC count, x10^3^/μL | 1.000 (1.000–1.000) | 0.855 |  |  |  |
| Neutrophil count, x10^3^/μL | 1.000 (1.000–1.000) | 0.554 |  |  |  |
| *Neutrophil > 3500 /μL* | 2.175 (0.942–5.026) | 0.069 |  |  |  |
| Lymphocyte count, x10^3^/μL | 1.000 (0.999–1.001) | 0.700 |  |  |  |
| *Lymphopenia (<1500/μL)* | 0.794 (0.326–1.929) | 0.610 |  |  |  |
| Platelet count, x10^3^/μL | 0.988 (0.980–0.996) | 0.003 |  |  |  |
| *Thrombocytopenia (<150 x10^3^/μL)* | 2.959 (1.297–6.751) | 0.010 |  | 1.909 (0.814–4.478) | 0.003 |
| Total bilirubin, mg/dL | 1.229 (0.268–5.626) | 0.791 |  |  |  |
| Albumin, g/dL | 0.389 (0.104–1.461) | 0.162 |  |  |  |
| *Albumin < 4.0 g/dL* |  |  |  |  |  |
| AST, IU/L | 1.004 (0.985–1.024) | 0.663 |  |  |  |
| ALT, IU/L | 0.999 (0.982–1.016) | 0.889 |  |  |  |
| BUN, mg/dL | 1.032 (0.943–1.129) | 0.494 |  |  |  |
| *BUN elevation (>19 mg/dL)* | 2.669 (0.908–7.848) | 0.074 |  |  |  |
| Creatinine, mg/dL | 2.558 (0.598–10.945) | 0.205 |  |  |  |
| *Creatinine elevation (>1.2 mg/dL)* | 1.223 (0.165–9.072) | 0.844 |  |  |  |
| CPK, IU/L | 1.001 (0.999–1.002) | 0.446 |  |  |  |
| *CPK elevation (>250 IU/L)* | 1.358 (0.318–5.790) | 0.679 |  |  |  |
| LDH, IU/L | 1.005 (1.002–1.008) | 0.001 |  | 1.004 (1.001–1.008) | 0.025 |
| *LDH elevation (>400 IU/L)* | 2.080 (0.918–4.716) | 0.079 |  |  |  |
| CRP, mg/dL | 1.351 (1.181–1.545) | 0.000 |  |  |  |
| *CRP elevation (>1.5 mg/dL)* | 4.667 (2.057–10.588) | 0.000 |  |  |  |
| *CRP elevation (>5 mg/dL)* | 9.392 (3.475–25.385) | 0.000 |  | 5.881 (1.843–18.771) | 0.003 |
| **Underlying diseases** |  |  |  |  |  |
| Cardiovascular disease | 1.679 (0.499–5.653) | 0.403 |  |  |  |
| Respiratory disease | 0.927 (0.125–6.880) | 0.941 |  |  |  |
| Diabetes mellitus | 1.001 (0.341–2.943) | 0.998 |  |  |  |
| Hypertension | 2.051 (0.905–4.649) | 0.085 |  |  |  |
| Charlson Comorbidity Index | 0.680 (0.284–1.628) | 0.386 |  |  |  |
| **Regdanvimab treatment** | 0.275 (0.102–0.740) | 0.011 |  | 0.176 (0.060–0.516) | 0.002 |

Abbreviations: HR, hazard ratio; CI, confidence interval; BMI, body mass index; Sx, symptom; Ct, cycle threshold; NP, nasopharyngeal; RdRp, RNA-dependent RNA polymerase; SIRS, systemic inflammatory response syndrome; SpO_2_, saturation of percutaneous oxygen; WBC, white blood cell; AST, aspartate aminotransferase; ALT, alanine aminotransferase; BUN, blood urea nitrogen; CPK, creatine phosphokinase; LDH, lactate dehydrogenase; CRP, C-reactive protein
